# Bt Exposure-Induced Death of *Dioryctria abietella* (Lepidoptera: Pyralidae) Involvement in Alterations of Gene Expression and Enzyme Activity

**DOI:** 10.3390/insects16101010

**Published:** 2025-09-28

**Authors:** Xiaomei Wang, Jiaxing Sun, Ya Xing, Ruting Chen, Defu Chi

**Affiliations:** 1 Key Laboratory for Sustainable Forest Ecosystem Management of Ministry of Education, College of Forestry, Northeast Forestry University, Harbin 150040, China; 2 Beidagang Wetland Nature Reserve Management Center, Tianjin 300270, China; 3 College of Mathematics and Computational Sciences, Tangshan Normal University, Tangshan 063000, China

**Keywords:** transcriptome, *Bacillus thuringiensis*, insecticidal activity, enzyme activity, differentially expressed genes, *Dioryctria abietella*

## Abstract

*Dioryctria abietella* (Lepidoptera: Pyralidae) is a destructive forest pest for coniferous trees. *Bacillus thuringiensis* has been widely applied in forestry as a biological control agent to control it. However, the mechanisms of Bt-induced mortality in *D. abietella*, particularly its effects on gene expression and enzyme activities, remain unclear. Here, bioassay, enzyme assay, transcriptome sequencing, and gene expression profiling were employed to explore the relationship between the toxin-receptor, defense, and lethal mechanisms of *D. abietella* after Bt exposure. In a toxicity bioassay, *Bacillus thuringiensis galleriae 05041* strain (*Bt05041*) was the most toxic insecticide to the larvae of *D. abietella,* with LC_50_ values of 3.15 × 10^8^ Colony-Forming Units (CFUs) mL^−1^ at 72 h after treatment. Transcriptome analysis revealed that the gene expression patterns of *D. abietella* after 8 h of *Bt05041* exposure (Bt8) varied considerably from the *Bt05041*-treated for 2 h group (Bt2). In the Bt2 group, differentially expressed genes were significantly enriched in cellular and bioenergy pathways of lysosome, insulin signaling, cGMP-PKG signaling, etc. Immune-related pathways were activated, namely cAMP, AMPK, MAPK, Rap1, IMD, and Toll pathways. Meanwhile, Bt8 treatment caused metabolic changes in basic substances such as amino acids, glucose, nucleic acids, and fatty acids. *Bt05041* exposure activated the activities of defense enzymes and induced gene expression changes in *D. abietella* larvae. Among them, most Bt-receptor genes had higher expression levels than defense enzyme genes. Overall, these findings reveal a possible mechanism underlying Bt-mediated death in *D. abietella* larvae. This work provides valuable information in terms of biological control strategies.

## Simple Summary

This study investigates the lethal effects of Bacillus thuringiensis (Bt) infection on *Dioryctria abietella* (Lepidoptera: Pyralidae) larvae. *D. abietella* is a destructive wood-boring pest that causes significant economic losses to Korean pine forests worldwide. Here, we tested larval survival, enzyme activity, and gene expression to explore the possible mechanism underlying Bt-mediated death in *D. abietella* larvae. Our findings demonstrate that Bt exposure significantly decreased the survival of *D. abietella* larvae and altered the activities of antioxidant and detoxification enzymes. Among them, *Bacillus thuringiensis galleriae 05041* strain (*Bt05041*) was the most toxic insecticide. Transcriptome analysis revealed that gene expression in *D. abietella* changed over time with *Bt05041* exposure, and high expression of toxin-receptors enhanced Bt’s insecticidal effect. These findings offer new insights for improving the biocontrol strategies of *D. abietella*.

## 1. Introduction

Globally, wood-boring pests have been one of the most devastating biotic factors in forestry production, and their persistent outbreaks have affected the functional services provided by forests, such as carbon storage, biodiversity habitat, and the availability of forest products, resulting in significant economic and ecological losses [1]. Classical biological control is a widely used strategy against wood-boring pests in Integrated Pest Management systems, while other tactics are not readily applicable in forests as a result of their long lifespan and complex structure [2,3]. Under this strategy, the entomopathogenic microorganisms dominated by *Beauveria bassiana*, *Bacillus thuringiensis*, and *Baculovirus* can achieve persistent pest suppression and bar the geographic spread of pests [2,4]. These natural pathogens, without harming natural enemies and non-target organisms, are major factors in reducing environmental risks [5].

*Bacillus thuringiensis* is a soil bacterium containing insecticidal crystal proteins (Crys) that are solubilized in the larval midgut to release toxins [6]. The binding of each toxin to receptors in the brush border membrane is a necessary step for toxicity, resulting in midgut perforation to trigger osmotic shock death [7,8]. Many research efforts have taken place in insect receptors that mediate toxicity, and four major types of receptors have been characterized: cadherin (Cad), aminopeptidase N (APN), alkaline phosphatase (ALP), and ATP-binding cassette transporter (ABC), which are proposed to be involved in the action of Cry toxins [9]. For example, Cry receptor genes (*Cad*, *ALP*, *APN1*, *ABCC2*) were substantially up-regulated in the midgut tissue of fourth-instar larvae upon early exposure to a sub-lethal dose of Cry1AcF toxin [10]. Likewise, the cadherin peptide (rTmCad1p) enhanced Cry3Aa toxicity, while midgut cadherin protein (PgCad1) caused by changes in amino acid sequence increased the resistance of pink bollworm *Pectinophora gossypiella* to Cry1Ac [11,12]. ABC family protein, HaABCA2, was observed to be a functional receptor of Bt Cry2AB toxin, and decreased expression of the *PxABCB2* gene slowed down the toxicity of diamondback moth *Plutella xylostella* to Cry1Ac [13,14]. ALPs play a key role in the toxicity of Cry1A, Cry2A, and Cry1C, and the knockout of *Csalp1*, *Csalp2*, and *Csalp4* effectively reduced larval mortality in the striped stem borer *Chilo suppressalis* [15]. In general, herbivorous pests after Bt-feeding can produce characteristic symptoms, including sluggishness, feeding cessation, paralysis, and body blackening, etc. Therefore, Bt has been widely used as a biopesticide worldwide against some species of Lepidoptera, Coleoptera and Diptera [16,17].

Differences in larval toxicity from Bt exposure might not only rely on toxin-receptor interaction, but also on host defense mechanisms [18]. When Bt violates the intestinal barriers, insects switch on the innate immune response [19]. Detoxification enzymes and antioxidant enzymes are important families of enzymes in the insect defense system and play important roles in insects’ tolerance to Bt. For example, different patterns of phenoloxidase activity were observed in the Colorado potato beetle *Leptinotarsa decemlineata* when larvae were attacked by Bt toxin [20]. In the silkworm *Bombyx mori*, the activity of both detoxification enzymes and antioxidant enzymes was increased by Cry1F exposure [21]. In contrast, the *PPO1* gene was down-regulated in *P. xylostella* at 18 h after Bt infection [22]. Moreover, lysosomes produced in the insect fat body can also act as a cationic defense peptide to play a detoxifying role [23].

*Dioryctria abietella* (Lepidoptera: Pyralidae) [24] is one of the most destructive wood-boring pests in forests, with populations mainly in China, Europe, and North America [25]. The larvae feed on the cone tissue of coniferous species, resulting in cone dysplasia and seed loss [26]. Although insects employ innate immune defenses involving defensive enzyme expression against microbial pathogens, Bt overcomes these defenses through targeted toxin production. The molecular mechanisms underlying Bt-induced mortality in *D. abietella* remain poorly understood. The objectives of this study were to examine the effects of toxin-receptors and defense enzymes on the death of *D. abietella* under Bt exposure. In this work, the toxicity of biocontrol strains to the *D. abietella* larvae was compared. The annotation function and metabolic pathways of differentially expressed genes (DEGs) after *Bacillus thuringiensis galleriae 05041* strain (*Bt05041*) exposure were analyzed using transcriptome technology. And a combination of enzyme assays and RT-qPCR was used to evaluate the expression Bt-mediated Cry receptors and immune responses. Taken together, the present work revealed the underlying mechanism of Bt-mediated mortality of *D. abietella*. This work will provide valuable information for deciphering the interactions between Bt and *D. abietella*, and it will benefit the development of new biological control strategies.

## 2. Materials and Methods

### 2.1. Insects and Pathogens

In August 2020, Korean pinecones with excrement were collected from the Yiqing Forest Farm in Yichun City, Heilongjiang Province, China (latitude 47.89° N, longitude 129.16° E). The damaged cones were dissected to obtain *D. abietella* larvae [26,27]. Insects were reared in the laboratory at 25 ± 1 °C, with a 16L:8D photoperiod and 50 ± 10% relative humidity. Larvae were fed on a modified semi-artificial diet [26] until the fifth instar for all experiments.

*Bacillus thuringiensis entomocidus 223176* strain (*Bt223176*) and *B. thuringiensis* sp. *2913* strain (*Bt2913*) were obtained from Bena Culture Collection Co., Ltd., Henan, China. *B. thuringiensis galleriae 05041* strain (*Bt05041*) was bought from Beijing Baio Bowei Biotechnology Co., Ltd., Beijing, China. Spore-crystal mixtures were cultivated on Luria–Bertani (LB) medium at 37 ± 1 °C for 72 h. Round, opaque, and off-white colonies were selected and incubated overnight at 37 ± 1 °C with 220 rpm in LB solution (peak of parasporal crystal production), followed by centrifugation at 12,000 rpm for 2 min [28]. The pellet was washed and collected in 0.1 M phosphate-buffered saline (PBS). The suspension of *Bt223176*, *Bt2913*, and *Bt05041* was adjusted to 1 × 10^8^ Colony-Forming Units (CFUs) mL^−1^ (OD_600nm_ = 0.5) and further diluted to 1 × 10^6^ and 1 × 10^4^ CFU mL^−1^.

### 2.2. Bioassays

Insect bioassays were referenced to the previously documented methodology [29]. Newly molted fifth instar of *D. abietella* were starved for 6 h and divided into two groups (treatment and control). The treatment group (*N* = 540) was further divided into nine subgroups with three replicates. In each subgroup, 12 mL of Bt solution (1 × 10^4^, 1 × 10^6^ and 1 × 10^8^ CFU mL^−1^ *Bt223176*; 1 × 10^4^, 1 × 10^6^ and 1 × 10^8^ CFU mL^−1^ *Bt2913*; 1 × 10^4^, 1 × 10^6^ and 1 × 10^8^ CFU mL^−1^ *Bt05041*) was added to 600 g of semi-artificial diet (50 °C) to feed 60 *D. abietella* larvae. In the control group (*N* = 60), 12 mL of 0.1 M PBS was added to 600 g of semi-artificial diet. All larvae were fed a semi-artificial diet for 10 days. Deaths were recorded every 2 days.

### 2.3. Enzyme Assays

As described in Section 2.2, 1080 larvae (216 larvae per time point with 24 treatments) were collected at 1 × 10^8^ CFU mL^−1^ concentrations for enzyme activity assays after 2, 6, 12, 24, and 48 h of exposure. Three biological replicates were performed for every treatment. Larval tissues were prepared using the method described by Singh et al. (2023) [30] with minor modifications. The enzyme activities were measured using the following assay kits, viz., Superoxide Dismutase (SOD) Assay Kit (G0101F, Suzhou Grace Biotechnology, Suzhou, China), Peroxidase (POD) Assay Kit (G0107F, Suzhou Grace Biotechnology, China), Catalase (CAT) Assay Kit (G0105F, Suzhou Grace Biotechnology, China), Glutathione S-transferase (GST) Assay Kit (G0208F, Suzhou Grace Biotechnology, China), phenol oxidase (PO) Assay Kit (G0146F, Suzhou Grace Biotechnology, China), and Acetylcholinesterase (AchE) Activity Assay kit (ACHE-2-W, Suzhou Comin Biotechnology, Suzhou, China), according to the manufacturer’s instructions.

### 2.4. RNA Extraction, cDNA Library Construction and Sequencing

As described in the bioassay of this article, 90 larvae (30 larvae per treatment with three replicates) were collected at 1 × 10^8^ CFU mL^−1^ concentrations after 2 and 8 h of *Bt05041*- exposure (taking 2 h after exposure as controls, abbreviated as CK). Total RNA was extracted from *D. abietella* larvae using a MiniBEST^TM^ Universal RNA Extraction Kit (9767, TaKaRa, Osaka, Japan) according to the manufacturer’s instructions. RNA purity and concentration were determined using a Qubit 2.0 Fluorometer (Invitrogen, Carlsbad, CA, USA) and integrity was determined via 1% agarose gel electrophoresis.

The eukaryotes have the structure of a ploy A tail at 3′-end, and mRNA was enriched from total RNA using magnetic beads with Oligo (dT) [31]. First strand cDNA was synthesized using random hexamer primers and reverse transcriptase (Invitrogen, USA), followed by second-strand cDNA synthesis under the catalysis of DNA polymerase I (TaKaRa, Japan). The cDNA was purified using the AMPure XP beads system (Beckman Coulter, Indianapolis, IN, USA). Selected cDNA fragments were enriched and amplified via PCR to construct cDNA libraries [32]. The cDNA libraries were sequenced using an Illumina^®^ HiSeq^TM^ X Ten (Sangon Biotech Co., Ltd., Shanghai, China) to generate paired-end reads. The raw sequence data were deposited in the National Center for Biotechnology Information’s (NCBI, https://www.ncbi.nlm.nih.gov/, accessed on 10 September 2024) Short Read Archive BioProject under the accession number PRJNA1159397.

### 2.5. De Novo Assembly and Gene Annotation

Raw data was filtered by removing the reads with adaptor content, N bases, and low quality to obtain clean data [33] using Trimmomatic 0.36 (http://www.usadellab.org/cms/?page=trimmomatic, accessed on 10 September 2024) [34]. Unigenes were generated by assembling clean reads using Trinity 2.4.0 (https://github.com/trinityrnaseq/trinityrnaseq/releases/tag/Trinity-v2.4.0, accessed on 10 September 2024) [35]. TPM (Transcripts Per Million reads) values were calculated for each sample to estimate expression levels between different genes [36]. Gene annotation was conducted for all unigenes using the NCBI databases, including the Conserved Domain Database (CDD, https://www.ncbi.nlm.nih.gov/cdd/, accessed on 10 September 2024), Eukaryotic Ortholog Groups (KOG, http://genome.jgi-psf.org/help/kogbrowser.jsf, accessed on 10 September 2024), Non-redundant Protein Sequence (NR, http://ncbi.nlm.nih.gov/), Protein Family (PFAM, http://pfam.xfam.org/, accessed on 10 September 2024), Nucleotide Sequences (NT, http://ncbi.nlm.nih.gov/, accessed on 10 September 2024), Kyoto Encyclopedia of Genes and Genomes (KEGG, https://www.kegg.jp/, accessed on 10 September 2024), and Gene Ontology (GO, http://www.geneontology.org/, accessed on 10 September 2024).

### 2.6. DEGs Analysis

The DEGs among the groups (Bt2 vs. CK, Bt8 vs. CK and Bt2 vs. Bt8) were identified using the DEseq2 1.12.4 (R language, https://bioconductor.org/packages/release/bioc/html/DESeq2.html, accessed on 10 September 2024) [37] with the thresholds of qValue ≤ 0.05 and |log2FC(Fold change)| ≥ 1. Functional annotation and enrichment analysis of DEGs were performed on the basis of the GO and KEGG databases. The gene expression in samples was observed using Venn diagrams, boxplots, and principal component analysis (PCA).

### 2.7. Gene Expression Analysis

RNA extraction and cDNA synthesis were performed from *D. abietella* as described by Xing et al. (2022) [25]. Quantitative reverse transcription PCR (qRT-PCR) was used to verify the relative expression levels of 14 DEGs (*DabiABCA2*, *DabiABCC1*, *DabiABCC5*, *DabiABCG1*, *DabiABCG3*, *DabiAPN4*, *DabiAPN7*, *DabiAPN8*, *DabiCad1*, *DabiSOD1*, *DabiSOD2*, *DabiGST5*, *DabiGST6,* and *DabiGST7*). qPCR primers were designed using Primer Premier 6.0 (Premier, Vancouver, BC, Canada) based on sequences obtained from the transcriptome, as shown in Table S2. qRT-PCR reactions were performed in a reaction mixture (50 μL) containing 25 μL of 2×UltraSYBR^®^ Mixture (CWBIO, Taizhou, China), 1 μL of each primer, 2 μL of cDNA template, and 21 μL of RNase-free water, using a CFX96 Touch^TM^ Real-Time PCR Instrument (BIORAD, USA) with cycling conditions set as follows: denaturing at 95 °C for 10 min, followed by 40 cycles of 95 °C for 15 s and 60 °C for 1 min. A melt curve was performed at the end of each reaction by increasing the incubation temperature from 60 °C in 0.5 °C increments to 95 °C with a 15 s dwell time to assess the specificity of a single amplicon. The *EF1α1* and *RPS3* were selected as housekeeping genes [25], and the relative expression of each gene was calculated using the 2^−ΔΔCt^ method [38].

### 2.8. Statistical Analysis

Data were presented as the mean ± standard error (S.E.) of three replicates and analyzed using one-way ANOVA and Tukey’s post hoc tests using SPSS 24.0 (IBM, Armonk, NY, USA). Data were involved in the following variables: larval survival, mortality, enzyme activity, gene expression level, GO function of DEGs, and KEGG enrichment of DEGs. Larval survival curves were drawn at concentrations of 1 × 10^8^ CFU mL^−1^ using GraphPad Prism 8 (GraphPad Software, La Jolla, CA, USA) and analyzed by the pairwise log-rank test for significance between treatment and control groups [39]. LC_50_ values (lethal concentration, 50% mortality) and the regression equation were calculated via probit analysis at concentrations of 1 × 10^8^ CFU mL^−1^ after 3 days of treatment (peak of larval death). The chi-square test was performed to determine whether a significant association exists between the concentration of Bt suspension and larval mortality of *D. abietella*. The cumulative mortality was calculated as follows: Cumulative mortality = (number of dead larvae within 10 days/number of test larvae) × 100%. The *p*-values of <0.05 and <0.01 were regarded to be significant and extremely significant, respectively.

## 3. Results

### 3.1. Toxicity of Bt Against D. abietella Larvae

**Figure 1:**
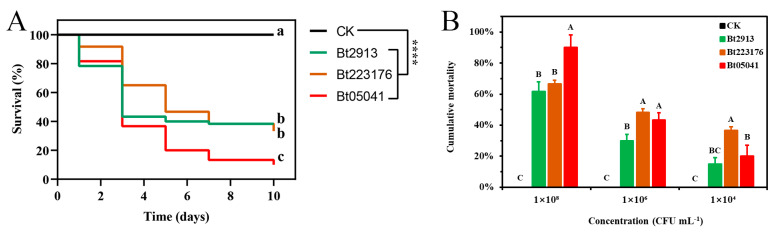
Bioassays of *D. abietella* larvae within 10 days after Bt infection. (**A**) Survival curves. Curves were drawn at concentrations of 1 × 10^8^ CFU mL^−1^. Different letters indicate significant differences among groups (*N* = 60 larvae per group, pairwise log-rank test, *p* < 0.05). **** indicates *p* < 0.0001 between Bt treatments and CK (pairwise log-rank test). (**B**) Larval mortality rates. Mortality rates are given as mean ± SE (*n* = 3). Different letters indicate significant differences among groups under the same concentration (*N* = 60 larvae per treatment, Tukey’s post hoc test, *p* < 0.01).

The three strains exhibit similar pathogenic mechanisms mediated by their insecticidal crystal proteins during the growth cycle yet display different virulence to *D. abietella* larvae. At a concentration of 1 × 10^8^ CFU mL^−1^, larval survival of *D. abietella* larvae rapidly decreased after Bt treatments compared to the controls (Figure 1A; log-rank test; *Bt2913*: *X*^2^ = 53.04, df = 1, *p* < 0.0001; *Bt223176*: *X*^2^ = 59.97, df = 1, *p* < 0.0001; *Bt05041*: *X*^2^ = 104.8, df = 1, *p* < 0.0001). The toxicity comparisons of different treatments are presented in Table 1. In all treatments, *Bt05041* had a significantly higher lethal effect, with LC_50_ values of 3.15 × 10^8^ CFU mL^−1^ (4.02 × 10^7^–2.98 × 10^10^; chi-square test: *X*^2^ = 1.897, df = 7, *p* = 0.000) and a cumulative mortality of 90.00 ± 8.16% after 3 days of treatment, which was significantly higher than other treatments (Figure 1B; ANOVA: *F*_3,8_ = 106.317, *p* < 0.01). In contrast, *Bt2913* and *Bt223176* exhibited comparable toxicity, inducing 62–67% mortality (Figure 1B). At the concentrations of 1 × 10^6^ CFU mL^−1^ and 1 × 10^4^ CFU mL^−1^, *D. abietella* larvae showed a mild pathogenicity after Bt-feeding but overall trends were significantly higher than the controls (Figure 1B; ANOVA; 1×10^6^: *F*_3,8_ = 84.792, *p* < 0.01; 1 × 10^4^: *F*_3,8_ = 25.308, *p* < 0.01).

### 3.2. Transcriptome Profiling Data

A total of 604,115,304 raw reads were generated. After quality control, approximately 566,739,834 clean reads with an average length of 142.17 bp were obtained. The Q30 was > 94%, the N percentage was ≤ 0.2%, and the average GC content = 48.48%, indicating that the sequencing data were accurate, high-quality fragments (Table 2). The assembly yielded 260,988 transcripts with an average length of 657.54 bp and the unigene dataset included 126,295 sequences. In total, 54,125 unigenes (42.86% of all unigenes) were annotated in at least one database by a BLAST 2.14.0 search, including 11,500 (9.11%) in CDD, 14,722 (11.66%) in KOG, 38,232 (30.27%) in NR, 12,780 (10.12%) in PFAM, 32,210 (25.5%) in NT, 9359 (7.41%) in KEGG, and 18,134 (14.36%) in GO (Table S1). Based on the species distribution of NR database, it was shown that the unigenes exhibited the highest homology to genes from the Navel orangeworm *Amyelois transitella* (52.13%), followed by the wax moth *Galleria mellonella* (5.7%), the Asian corn borer *Ostrinia furnacalis* (4.65%), and the cotton bollworm *Helicoverpa armigera* (4.36%) (Figure S1).

### 3.3. DEGs After Bt05041 Exposure in D. abietella

**Figure 2:**
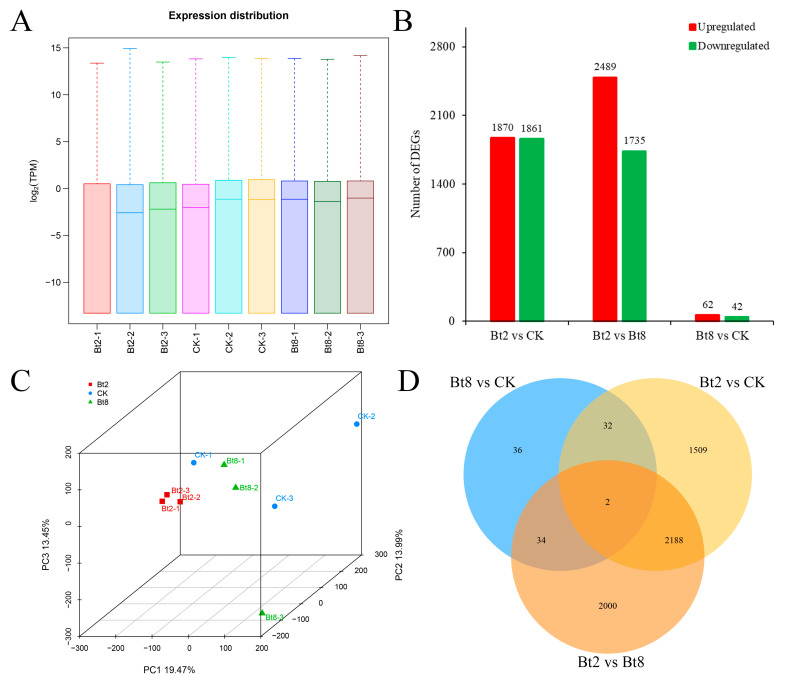
The DEGs in *D. abietella* larvae exposed to *Bt05041* treatment after different numbers of days. (**A**) Boxplots of gene expression based on the TPM values in samples (*n* = 3). (**B**) The number of DEGs in comparison groups. (**C**) Principal component analysis (PCA) of gene expression in samples (*n* = 3). (**D**) Venn diagram of shared and unique DEGs. CK, control 2 h; Bt2, *Bt05041* treatment for 2 h; Bt8, *Bt05041* treatment for 8 h.

The distribution of gene expression under Bt05041 treatments was evaluated based on TPM values (Figure 2A). The gene expression levels slightly fluctuated in the Bt2 treatment group, but the trend was basically consistent and reproducible among all samples. Principal component analysis showed that the samples in the CK and Bt8 groups were relatively dispersed, but the samples in Bt2 group were tightly clustered together (Figure 2C). A total of 3731 (1870 up- and 1861 down-regulated), 4224 (2489 up- and 1735 down-regulated), and 104 (62 up- and 42 down-regulated) DEGs were identified in the Bt2 vs. CK, Bt2 vs. Bt8, and Bt8 vs. CK groups, respectively (Figure 2B). The number of DEGs in the Bt8 vs. CK group was much lower than that of the Bt2 vs. CK and Bt2 vs. Bt8 groups. Consistently, a Venn diagram showed that the Bt8 vs. CK group (36) had uniquely fewer DEGs than the Bt2 vs. CK (1500) and Bt2 vs. Bt8 (2000) groups (Figure 2D). These results demonstrate that the gene expression patterns of *D. abietella* after 2 h of Bt exposure varied considerably from that in *D. abietella* between the controls and 8 h of Bt exposure.

In the GO database, all DEGs were classified into three categories: biological process, cellular component, and molecular function (Figure S2A–C). In the category of biological process, cellular process and metabolic process occupied the main position in the Bt2 vs. CK, Bt8 vs. CK, and Bt2 vs. Bt8 groups. In the cell components branch, cell and cell part were the two maximal categories in the Bt2 vs. CK, Bt8 vs. CK, and Bt2 vs. Bt8 groups. In the molecular function component, binding, catalytic activity, and transporter activity were the three maximal categories in the Bt2 vs. CK, Bt8 vs. CK, and Bt2 vs. Bt8 groups. This result indicates that *Bt05041* exposure activated the immune system in *D. abietella* and triggered the expression of DEGs in cellular and metabolic processes.

**Figure 3:**
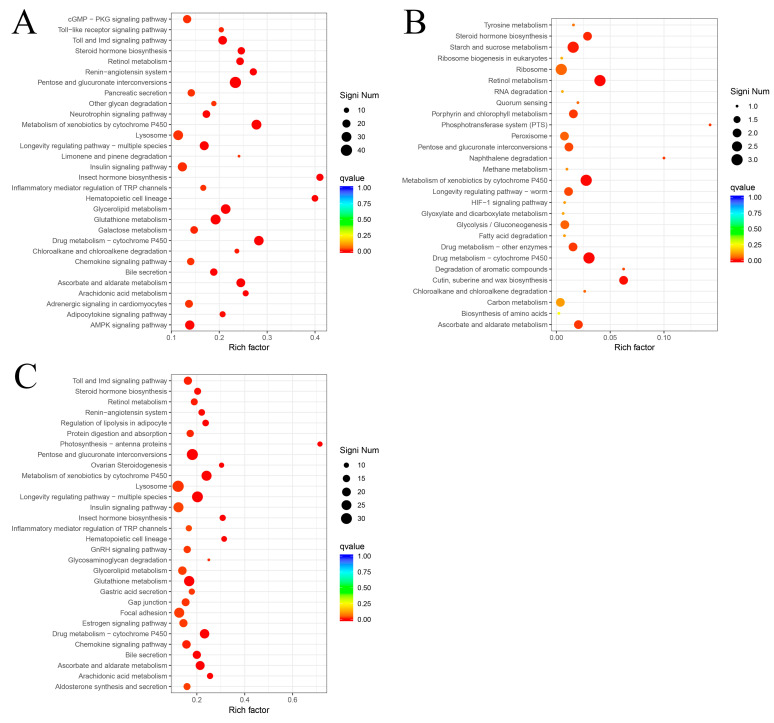
KEGG enrichment pathways of DEGs in *D. abietella* larvae at different days post-*Bt05041* exposure. (**A**) The top 30 most significant KEGG pathways in the Bt2 vs. CK group. (**B**) The top 30 most significant KEGG pathways in the Bt8 vs. CK group. (**C**) The top 30 most significant KEGG pathways in Bt2 vs. Bt8 group. The *x*-axis and *y*-axis represent the Rich factor and the KEGG pathway, respectively.

In the KEGG enrichment analysis, the most significantly enriched pathway of DEGs was lysosome in the Bt2 vs. CK group, followed by pathways related to the insulin signaling pathway, cGMP-PKG signaling pathway, AMPK signaling pathway, and galactose metabolism (Figure 3A). The four pathways “Ribosome”, “Carbon metabolism”, “Glycolysis/gluconeogenesis”, and “Peroxisome” were highly enriched with DEGs in the Bt8 vs. CK group (Figure 3B). At the same time, the DEGs in the Bt2 vs. Bt8 group were significantly different in the “Lysosome”, “Insulin signaling pathway”, “Focal adhesion”, and “Glycerolipid metabolism” pathways (Figure 3C). Under short-term stress of *Bt05041*, the DEGs were more concentrated in the cell digestive function and bioenergy generation, exchange and storage. Long-term exposure of *Bt05041* destroyed the structure in *D. abietella* cells [26], causing metabolic changes in basic substances such as amino acids, glucose, nucleic acids, and fatty acids. Moreover, to explore the DEGs affected by *Bt05041*-stress, we analyzed the up- and down-regulated genes in immune-related signaling pathways among the Bt2 vs. CK, Bt8 vs. CK, and Bt2 vs. Bt8 groups (Table 3). In the Bt2 vs. CK and the Bt2 vs. Bt8 groups, up-regulated genes were mainly enriched in “cAMP”, “Rap1”, and “MAPK” signaling pathways. The “AMPK” pathway was most enriched with down-regulated genes. The “p53”, “JAK-STAT” and “Toll” pathways were enriched for only the up-regulated genes. For the Bt8 vs. CK group, no enrichment of DEGs was found in the *Bt05041*-related signaling pathway. Therefore, it could be observed that insecticidal proteins were quickly recognized by pattern recognition receptors at 2 h after *Bt05041* exposure and activated the immune-related signaling pathway.

To validate the RNA-seq results, five DEGs (up-regulated in the Bt2 vs. CK group) were randomly selected to determine the expression levels by qRT-PCR. The results demonstrate that expression patterns were generally consistent between RNA-seq and qRT-PCR, showing similar trends (Figure S3).

### 3.4. Bt Exposure-Induced Immune Responses of D. abietella

**Figure 4:**
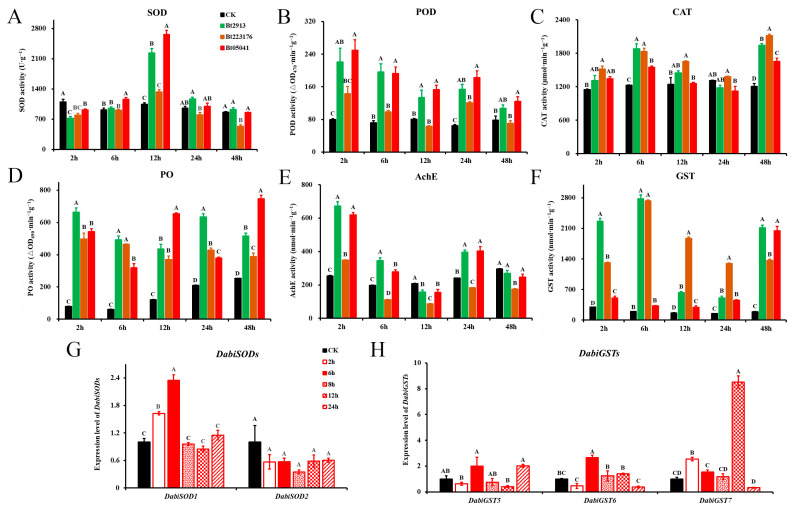
Bt exposure activated the activity of defensive enzymes and induced the gene expression of their genes. Enzyme activities of (**A**) SOD, (**B**) POD, (**C**) CAT, (**D**) PO, (**E**) AchE, and (**F**) GST after *Bt2913*, *Bt223176*, and *Bt05041* treatment. Expression levels of (**G**) superoxide dismutase genes and (**H**) glutathione-S-transferase genes after feeding *Bt05041* at different time points. Data are given as mean ± SE (*n* = 3). Different letters above bars indicate significant differences between time points (Tukey’s post hoc test, *p* < 0.01).

The response of antioxidant enzymes-related genes (SOD, POD, CAT, and PO), detoxification enzymes-related genes (AchE and GST) and Bt-receptor genes was evaluated after Bt exposure. There was a significant increase at 12 h after Bt treatment in SOD activity compared to the control (Figure 4A; ANOVA: *F*_3,8_ = 197.855, *p* < 0.01). The SOD genes exhibited an interesting expression pattern after *Bt05041* treatment. Compared with the control, the expression level of *DabiSOD1* was significantly up-regulated (Figure 4G; ANOVA: *F*_5,12_ = 105.833, *p* < 0.01), while the expression level of *DabiSOD2* remained basically unchanged (Figure 4G; ANOVA: *F*_5,12_ = 2.967, *p* = 0.057). The Bt exposure induced a significant increase in GST activity, which was significantly higher than the control at different time points (Figure 4F; ANOVA; 2 h: *F*_3,8_ = 977.971, *p* < 0.01; 6 h: *F*_3,8_ = 2006.937, *p* < 0.01; 12 h: *F*_3,8_ = 1772.143, *p* < 0.01; 24 h: *F*_3,8_ = 933.149, *p* < 0.01; 48 h: *F*_3,8_ = 436.933, *p* < 0.01). The three Bt strains elicited different insecticidal responses in *D. abietella* larvae. Among them, GST activity was 14.05 times higher than the control at 6 h after *Bt2913* treatment and reached a peak at 48 h after *Bt05041* treatment. Figure 4H shows the expression results of GST genes. *DabiGST5* expression was dramatically enhanced at 6 and 24 h when larvae were exposed to *Bt05041* (2.01-fold; ANOVA: *F*_5,12_ = 9.332, *p* < 0.01). Likewise, *DabiGST6* expression had a similar effect (2.65-fold at 6 h; ANOVA: *F*_5,12_ = 35.455, *p* < 0.01). Notably, the expression level of *DabiGST7* in *Bt05041*-exposed larvae showed an approximately 8.51-fold increase for 12 h (ANOVA: *F*_5,12_ = 320.629, *p* < 0.01). In other indicators, PO activity was significantly stimulated when larvae were exposed to *Bt2913* for 2 h (Figure 4D; 8.38-fold; ANOVA: *F*_3,8_ = 206.811, *p* < 0.01). In contrast, the activities of POD, CAT, and AchE were slightly increased after Bt exposure at different time points (Figure 4B,C,E). These results indicate that Bt exposure had a significant stimulatory effect on the activities of GST and PO and had strong induction of *DabiGST7* expression in *D. abietella* larvae.

**Figure 5:**
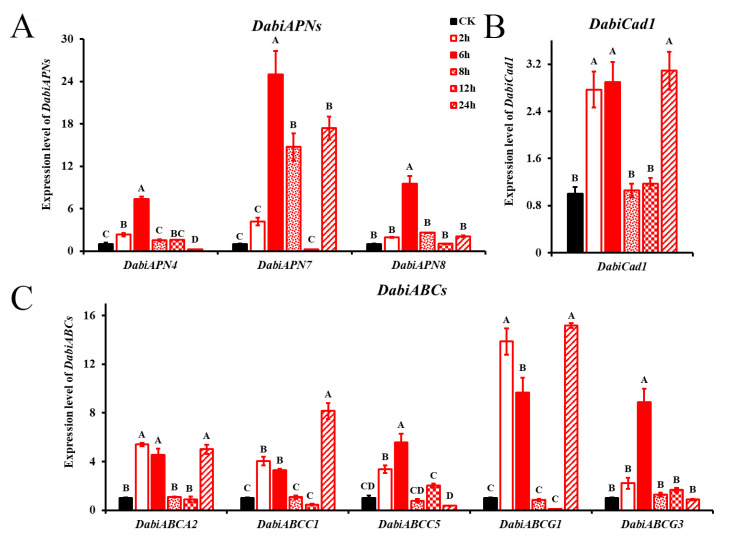
Effects of *Bt05041* on toxin-receptor genes expression in *D. abietella* larvae. (**A**) *DabiAPNs*. (**B**) *DabiCad1*. (**C**) *DabiABCs*. Data are given as mean ± SE (*n* = 3). Different letters above bars indicate significant differences between time points (Tukey’s post hoc test, *p* < 0.01).

APNs, Cads and ABCs are the main receptors to mediated Bt toxicity. As analyzed by qRT-PCR, larvae exposed to *Bt05041* at 6 h showed strong induction and increased the expression levels of *DabiAPN4*, *DabiAPN7*, and *DabiAPN8* by 7.36-, 24.96- and 9.56-fold, respectively (Figure 5A; ANOVA; *DabiAPN4*: *F*_5,12_ = 327.559, *p* < 0.01; *DabiAPN7*: *F*_5,12_ = 68.225, *p* < 0.01; *DabiAPN8*: *F*_5,12_ = 106.731, *p* < 0.01). Similarly, larvae exposed to *Bt05041* showed significantly increased expression levels of *DabiCad1* at three time points (2.77-fold at 2 h, 2.90-fold at 6 h and 3.09-fold at 24 h) (Figure 5B; ANOVA: *F*_5,12_ = 35.371, *p* < 0.01). Expression patterns of *DabiABCA2*, *DabiABCC1*, and *DabiABCG1* showed similar trends (Figure 5C). The expression levels of *DabiABCA2*, *DabiABCC1*, and *DabiABCG1* were significantly increased when fifth-instar *D. abietella* were exposed to *Bt05041* for 2, 6, and 24 h (ANOVA; *F*_5,12_ = 132.162, *p* < 0.01, 5.41-, 4.56-, 5.01-fold for *DabiABCA2*; *F*_5,12_ = 174.513, *p* < 0.01, 4.04-, 3.30-, 8.17-fold for *DabiABCC1*; *F*_5,12_ = 214.193, *p* < 0.01, 13.87-, 9.67-, 15.18-fold for *DabiABCG1*). Furthermore, larvae exposed to *Bt05041* at 6 h had a 5.58-fold increase for *DabiABCC5* and an 8.90-fold increase for *DabiABCG3* (Figure 5C; ANOVA; *DabiABCC5*: *F*_5,12_ = 67.498, *p* < 0.01; *DabiABCG3*: *F*_5,12_ = 82.508, *p* < 0.01). In summary, the expression of both defense enzyme genes and Bt-receptor genes in *D. abietella* larvae was significantly elevated, predominantly occurring at 6 h after Bt treatment; however, the expression level of Bt-receptor genes was substantially higher than those of defense enzyme genes, which likely contributed to the mortality of *D. abietella*.

## 4. Discussion

Based on validated efficacy and the development of sustainable agriculture, Bt is one of the most promising biopesticides in the field of pest control. Studies have demonstrated significant variation in insecticidal activity among different Bt strains, with pathogenicity determined by strain-specific Cry toxins and their interactions with target insect populations at specific developmental stages [40,41]. For example, all field-collected populations were more susceptible to Cry1Ab protein than the laboratory-adapted populations among geographically distinct populations of the southwestern corn borer *Diatraea grandiosella* [42]. The LC_50_ values reached 5.13 and 0.49 μg/mL when the northern corn rootworm *Diabrotica barberi* neonates were exposed to different concentrations of mCry3A and eCry3.1Ab [43], while acute tests estimated a 48 h-LC_50_ of 1850 μg/mL in Bt against the fifth instar of the aquatic insect *Chironomus riparius* [44]. The larval survival of larvae fed on artificial diets supplemented exclusively with Cry23Aa was approximately 69% in the cotton boll weevil *Anthonomus grandis*, while the combined provision of Cry23Aa and Cry37Aa toxins in the artificial diet led to larval mortality rates approaching 100% [45]. Our research showed that mortality rates of *D. abietella* larvae significantly elevated with increased concentrations after Bt exposure (Figure 1B). Among them, *Bt05041* exhibited greater toxicity (LC_50_ = 3.15 × 10^8^ CFU mL^−1^) than *Bt2913* (LC_50_ = 6.52 × 10^9^ CFU mL^−1^) at 72 h post-treatment (Table 1). These results demonstrate the different pathogenicity of different Bt strains to *D. abietella* larvae. Consistent with our study, a large number of studies have found that Bt was effective in controlling a variety of pests. For example, Bt spraying in the field was highly effective against the box tree moth *Cydalima perspectalis*, reducing larval density by more than 90% in all sites and years at 5 days after treatment [46]. Almost all early-instar monarch butterfly larvae (*Danaus plexippus*) were moribund or dead within 48 h of being Bt-sprayed and showed symptoms of Bt pathogenesis [47]. The corrected mortality of the second-instar red palm weevil (*Rhynchophorus ferrugineus*) larvae increased significantly after Bt-feeding, which could effectively reduce the damage to palm trees [48]. Moreover, environmental contaminants such as microplastics and heavy metals can alter the Bt susceptibility of insects [39,49]. The synergistic effect of environmental contaminants and Bt on larval mortality was not analyzed in this study.

It has been reported that Bt toxins induce differential expression in functional genes related to cell parts, cellular processes, binding, oxidation-reduction, and metabolic processes, resulting in serious damage to insect midgut cells [50,51]. The study revealed that the Bt2 vs. CK group exhibited more unique DEGs than the Bt8 vs. CK group (Figure 2D). This temporal pattern aligns with findings by Chen et al. (2024) [52], who observed more DEGs at 12 h than at 24 h in *D. abietella* larvae infected with *Bt 2913*. Consistent with these findings, Wang et al. (2023) [26] observed a significant reduction in gut microbial abundance between 3 and 9 h post-*Bt05041* exposure in *D. abietella*, accompanied by severe midgut epithelial damage including microvilli detachment, organelle deformation, cytoplasmic vacuolization, and nuclear fragmentation. These findings demonstrate that Bt crystal proteins exert transient insecticidal activity, initially inducing midgut epithelial perforation without causing immediate mortality. The primary tissue damage subsequently enables the proliferation of opportunistic pathogens, ultimately leading to lethal sepsis [53]. This pathogenic mechanism also explains the observed sharp decline in DEGs at 8 h post-*Bt05041* exposure. Based on the GO database, this study illustrated that *Bt05041* exposure activated the immune system of *D. abietella* larvae and triggered the expression of DEGs in cellular and metabolic processes (Figure S2). In KEGG enrichment analysis, short-term stress of Bt (Bt2) stimulated the cellular digestion function and bioenergy metabolism, and genes related to lysosome, the insulin signaling pathway, the cGMP-PKG signaling pathway, and the AMPK signaling pathway were significantly enriched (Figure 3A). Long-term exposure to Bt (Bt8) induces more defense mechanisms, leading to changes in many basic substances such as melanin synthesis, amino acids, and glucose metabolism (Figure 3B). Interestingly, there are significant differences between two groups (Bt2 vs. Bt8) in the “lysosome”, “insulin signaling pathway”, “focal adhesion”, and “glycerol metabolism” pathways (Figure 3C), suggesting that Bt toxins affect the function of intracellular proteins and the metabolism of fatty acids and glycerol, destroying the structure of *D. abietella* cells [26]. Many studies have also demonstrated the effects of Bt on these metabolic pathways. Specifically, Chen et al. (2024) [52] identified that a large number of unigenes were annotated to functional pathways such as signal transduction, translation, and energy metabolism in the midgut tissues of *D. abietella* larvae treated with *Bt 2913*. Xu et al. (2024) [54] reported that up-regulated differentially expressed proteins by the cotton leaf worm *Spodoptera litura* were mainly associated with neurodegenerative disease-related, lysosome, and oxidative phosphorylation pathways after Cry1Ab exposure. The down-regulated enrichment pathways were mainly related to ribosome and drug metabolism. How et al. (2024) [55] suggested that Bt infection caused significant enrichment of lysosomes and metabolic pathways in the nematode *Caenorhabditis elegans*. Wang et al. (2023) [51] confirmed that several lipid-related pathways involved in the response to Cry9A and Vip3A single and combined treatment for 6 h, such as glycerolipid, glycerophospholipid, and ether lipid metabolism. In addition, various pore-forming toxins activated the MAPK, Toll, IMD, JNK, and JAK-STAT signaling pathways in different organisms [22,56]. In this study, genes 2 h after Bt exposure were significantly enriched in immune-related signaling pathways such as cAMP, AMPK, MAPK, Rap1, IMD, and Toll (Table 3). This suggests that Bt ingestion rapidly activates complex defense responses in *D. abietella* larvae.

Bt toxin-receptor interaction and host immunity are key factors in the pathogenicity of insect populations. On the one hand, Bt strains elicit different insecticidal responses in insects, activating immune defenses through the upregulation of defense enzyme-related genes in multiple signaling pathways (cAMP, AMPK, MAPK, Rap1, IMD, and Toll) and subsequent induction of enzymatic activity. For instance, Bt-infected larvae triggered the prophenoloxidase cascade, and PO activity was significantly increased in the larvae of *C. riparius* and *G. mellonella* [44,57]. Activation of GST activity in the *L. decemlineata* hemolymph was shown on the 3rd day of Bt infection [58]. The activities of some antioxidant enzymes (SOD, POD, CAT) and some detoxification enzymes (AChE, P-450, CarE, GST) in the dark black chafer *Holotrichia parallela* larvae showed an activation–inhibition trend throughout the time course of nematode and Bt exposure [59]. The POD activity in Cry1F-exposed silkworms reached maximum levels (1750 U/g) at 48 h post-treatment, followed by a gradual decline [21]. Our data demonstrated that exposure to different Bt strains elevated both antioxidant (SOD, POD, CAT, PO) and detoxification (AChE, GST) enzyme activities in *D. abietella* larvae. *Bt05041* showed the strongest stimulant effect, significantly enhancing GST and PO activities while upregulating the expression of *DabiSOD1* and *DabiGST7* (Figure 4). On the other hand, the insecticidal specificity of Bt toxins is mediated by differential midgut receptor expression, subsequently inducing distinct gene expression profiles in target insects [60,61]. Ren et al. (2014) [62] significantly reduced the corresponding gene expression and decreased Cry1Ca-induced mortality in the beet armyworm *Spodoptera exigua* larvae after RNA interference with six *APNs*. Down-regulation of the ABC transporter gene *Pxwhite* contributed to high-level resistance to Bt Cry1Ac toxin in *P. xylostella* [63]. The cadherin gene *PgCad1* showed significantly higher expression in toxin-sensitive pink bollworm populations (APHIS-S) compared to resistant populations (APHIS-R) following Cry1Ac exposure [12]. Toxin-receptors (*ABCC2*, *Cad*, *ALP*, *APN1*) were substantially up-regulated in the midgut tissue of fourth-instar *G. mellonella* larvae upon early exposure (6 h) to Cry1AcF toxin [10]. The gene expression pattern was also obtained in *D. abietella* larvae, i.e., toxin-receptors (*DabiAPN4*, *DabiAPN7*, *DabiAPN8*, *DabiCad1*, *DabiABCA2*, *DabiABCC1*, *DabiABCC5*, *DabiABCG1*, and *DabiABCG3*) were significantly up-regulated after *Bt05041* exposure (Figure 5). Remarkably, we found that the elevated expression of the defense enzymes and Bt-receptor genes mostly occurred at 6 h after Bt treatment, but the expression levels of toxin-receptors were much higher than defense enzyme genes, which may have contributed to the large number of larval deaths.

## 5. Conclusions

This study demonstrated that Bt exposure significantly decreased the survival of *D. abietella* larvae and altered the activities of antioxidant and detoxification enzymes. Their exposure activated cAMP, AMPK, MAPK, Rap1, IMD, and Toll signaling pathways, and most DEGs related to cell digestion and fatty acids metabolism pathways were significantly enriched. Likewise, there were remarkable changes in the gene expression of *D. abietella* larvae, i.e., the up-regulation of GST, APN, and ABC transporter genes. Therefore, this study concluded that Bt toxins accelerated larval death by disrupting cell structure and disordering metabolic function, and the high expression of toxin-receptors improved insecticidal activity in *D. abietella*. In conclusion, this study reveals the mechanism underlying Bt-mediated death of *D. abietella* and provides a novel understanding for the development of biocontrol strategies.

## Figures and Tables

**Table 1:** Oral toxicity of Bt strains to *D. abietella* larvae after 72 h of treatments based on a chi-square test.

Treatment	*N*	Slope ± SE	*X* ^2^	df	*R* ^2^	LC_50_ (CFU mL^−1^)	95% CL	*p* Value
*Bt2913*	180	0.231 ± 0.069	1.757	7	0.972	6.52 × 10^9^	2.19 × 10^8^–7.65 × 10^14^	0.001
*Bt05041*	180	0.293 ± 0.068	1.897	7	0.965	3.15 × 10^8^	4.02 × 10^7^–2.98 × 10^10^	0.000
*Bt223176*	180	0.086 ± 0.065	0.609	7	0.999	1.62 × 10^15^	-	0.184

Notes: *N*, larvae number; SE, standard error; *X*^2^, chi-square; df, degree of freedom; *R*^2^, fitting goodness; LC_50_, lethal concentration at 50% mortality; CL, confidence limit; *p* value represents the statistical differences within the group.

**Table 2:** Statistics of *D. abietella* transcriptome in different samples.

Sample	Raw Reads	Clean Reads	Q20(%)	Q30(%)	GC(%)	N Percentage (%)	Total Mapping (%)	Uniquely Mapping (%)
Bt2-1	63,064,626	48,014,480	98.61	95.09	49.27	0.00	84.44	26.22
Bt2-2	60,253,896	54,363,680	98.74	95.56	51.42	0.00	88.11	22.86
Bt2-3	154,500,618	148,944,106	98.57	94.90	47.28	0.00	80.38	25.80
Bt8-1	51,279,414	49,466,618	98.52	95.12	48.39	0.20	88.95	24.96
Bt8-2	60,099,620	58,089,538	98.55	95.17	47.83	0.20	89.15	24.14
Bt8-3	50,968,208	49,377,024	98.62	95.38	48.21	0.20	89.01	24.78
CK-1	48,258,260	46,652,676	98.59	95.27	47.94	0.20	89.34	23.60
CK-2	59,812,572	57,894,058	98.61	95.35	47.89	0.20	89.16	24.29
CK-3	55,878,090	53,937,654	98.55	95.19	48.09	0.20	89.13	24.01

Notes: CK, control 2 h; Bt2, *Bt05041* treatment for 2 h; Bt8, *Bt05041* treatment for 8 h. Each treatment contained three independent replicates.

**Table 3:** The immune-related pathways in the *D. abietella* larvae-based KEGG database.

Pathways	Bt2 vs. CK			Bt2 vs. Bt8			Bt8 vs. CK		
	Up	Down	Total	Up	Down	Total	Up	Down	Total
**Signaling pathway**									
p53	4	0	4	2	0	2	0	0	0
AMPK	10	9	19	12	9	21	0	0	0
MAPK	14	1	15	19	0	19	0	0	0
Rap1	20	0	20	21	1	22	0	0	0
cAMP	22	3	25	24	2	26	0	0	0
JAK-STAT	9	0	9	8	0	8	0	0	0
Toll	12	0	12	9	0	9	0	0	0
Imd	11	1	12	10	0	10	0	0	0
**Pattern recognition receptors**									
NOD-like receptors	7	3	10	6	0	6	0	0	0
Toll-like receptors	9	1	10	7	0	7	0	0	0
RIG-I-like receptors	3	1	4	4	0	4	0	0	0
C-type lectin receptors	14	1	15	11	0	11	0	0	0

Notes: CK, control 2 h; Bt2, *Bt05041* treatment for 2 h; Bt8, *Bt05041* treatment for 8 h.

## Data Availability

The data presented in this study are openly available in [NCBI Short Read Archive BioProject] at [http://www.ncbi.nlm.nih.gov/bioproject/1159397, accessed on 10 September 2024], reference number [PRJNA1159397].

## Supplementary Materials

The following supporting information can be downloaded at https://www.mdpi.com/article/10.3390/insects16101010/s1, Figure S1: NR annotation distribution among other species of *D. abietella* larvae; Figure S2: GO function classification of DEGs in *D. abietella* larvae exposed to *Bt05041* treatment after different numbers of days. (A) Bt2 vs. CK. (B) Bt8 vs. CK. (C) Bt2 vs. Bt8. On the *x*-axis, GO terms were classified into three categories: biological process, cellular component, and molecular function. On the *y*-axis, light colors represent the number of DEGs and dark colors represent the percentage of DEGs in each subcategory. CK, control 2 h; Bt2, *Bt05041* treatment for 2 h; Bt8, *Bt05041* treatment for 8 h; Figure S3: Validation of RNA-seq expression profiles of DEGs by qRT-PCR in Bt2 vs. CK group; Table S1: Functional annotation of *D. abietella* transcriptome; Table S2: Primers designed for quantitative real-time PCR.
