# Supplementary material for: Bt Exposure-Induced Death of Dioryctria abietella (Lepidoptera: Pyralidae) Involvement in Alterations of Gene Expression and Enzyme Activity
Source: Insects. 2025 Sep 28;16(10):1010. doi: 10.3390/insects16101010 (PMC12563068; doi:10.3390/insects16101010)
Supplement: Supplementary file 1 [file insects-16-01010-s001.zip › insects-3735872-supplementary.pdf]

Figures

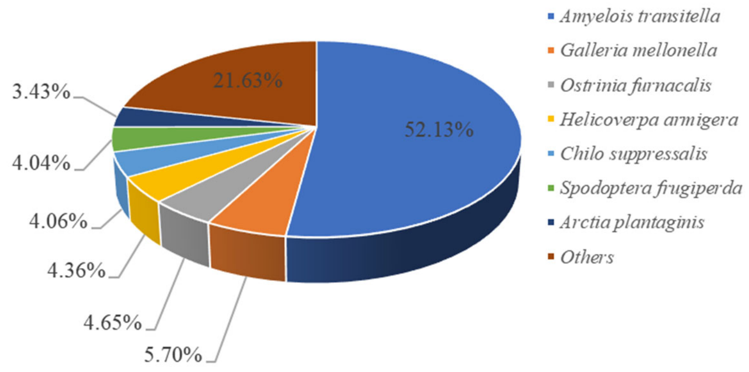

Figure S1 NR annotation distribution among other species of *D. abietella* larvae.

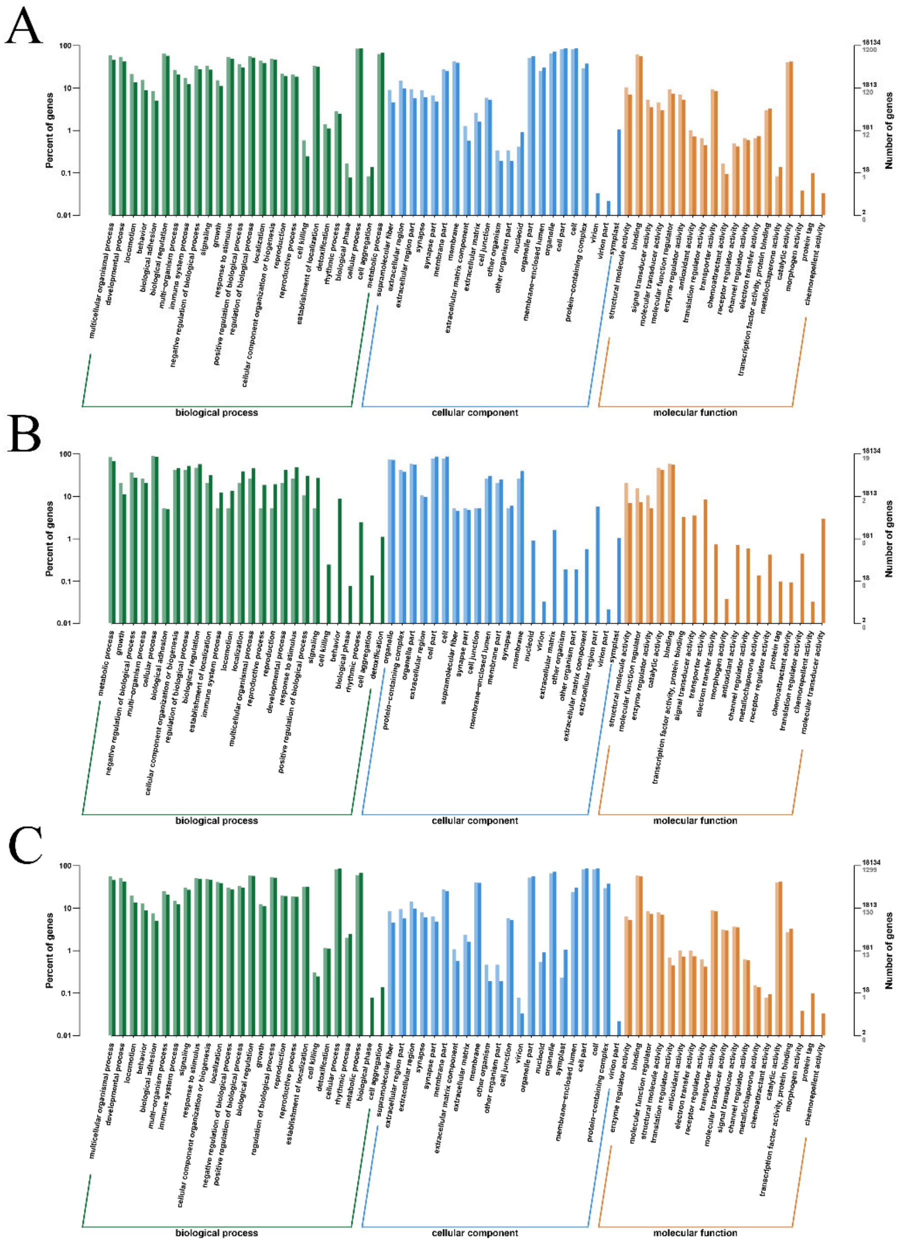
A
B
C
Percent of genes
Number of genes
biological process
cellular component
molecular function

Figure S2 GO function classification of DEGs in *D. abietella* larvae exposed to different days after Bt05041-treatment. (**A**) Bt2 vs CK. (**B**) Bt8 vs CK. (**C**) Bt2 vs Bt8. On the *x*-axis, GO terms were classified into three categories: biological process, cellular component, and molecular function. On the *y*-axis, light colors represent the number of DEGs and dark colors represent the percentage of DEGs in each subcategory. CK, control 2 h; Bt2, Bt05041 treatment for 2 h; Bt8, Bt05041 treatment for 8 h

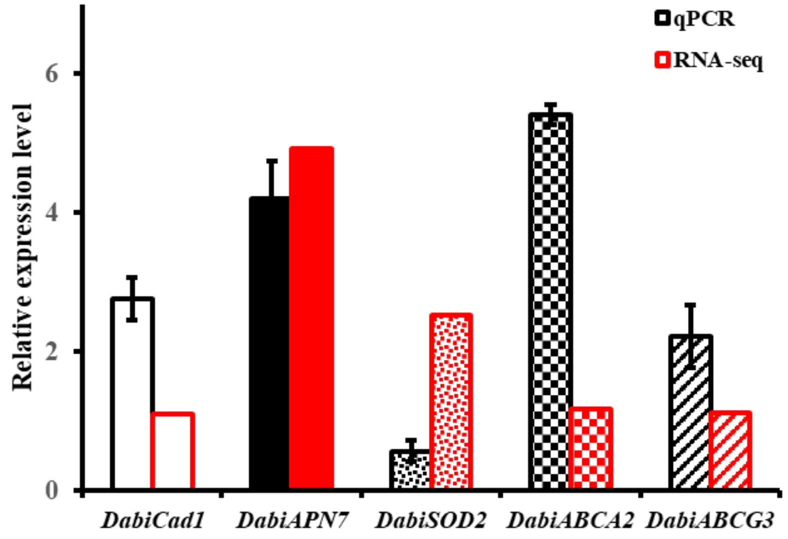

Figure S3 Validation of RNA-seq expression profiles of DEGs by qRT-PCR in Bt2 vs CK group.

Tables

Table S1. Functional annotation of *D. abietella* transcriptome.

Database	Number of genes	Percentage(%)
NT	32210	25.5
NR	38232	30.27
GO	18134	14.36
KOG	14722	11.66
KEGG	9359	7.41
PFAM	12780	10.12
CDD	11500	9.11
All	1532	1.21
At least one database	54125	42.86
Total genes	126295	100

Table S2. Primers designed for quantitative real-time PCR.

Gene name	Accession number	Forward primer (5´-3´)	Reverse primer (5´-3´)	Product size (bp)
**ATP-binding cassette sub-family**				
DabiABCA2	OP485302	GACTGCTGATGTGGAAGAA	CTGGAGAATATGCGATTGAC	240
DabiABCC1	OP485315	TTGCTGGAAGAGGAGGAA	GCGGAAGTAAGTGCTGTA	204
DabiABCC5	OP485317	TCTTGAGCAGGTGGAACT	GGATTGTCGTCTGAATGAAC	200
DabiABCG1	OP485318	GTTAAGTTCGGCGTCATCT	AAGGCGTAGTGGCAGTAA	212
DabiABCG3	OP485304	GGTTCTGCTACGCTTGTAT	GGCTGAGAGGTGATTGGA	217

**Aminopeptidase N**				
DabiAPN4	OP485299	GAACTATGACGATACCAACTG	CTACGGACGAAGGCTAATC	231
DabiAPN7	OP485314	TGATGAGCCTGTCTGATTC	CAAGTGCGAGATGTATGAAG	225
DabiAPN8	OP485301	ATCACACCAACAGCAACTA	GTCAGCCAGCAGAGATAC	227
**Cadherin**				
DabiCad1	OP485297	CCGACAGGAAGAAGATGG	CTAAGGAGCAGGAGTTCAG	194
**Superoxide dismutase**				
DabiSOD1	OP485305	CGGTGAATCAAGAGTAGCA	GCTCGTGGATGTGGAATC	164
DabiSOD2	OP485320	TTAGAGATGTTGCCGAAGTT	AGAGCGAAGTCCAATAACG	188
**Glutathione S-transferase**				
DabiGST5	OP485324	GGAGTTGCCGAACCAATT	GACATGACGCAGACGAAT	166
DabiGST6	OP485308	TTCTTCCTTAGCGAGAGTAAC	GCACTGATGTTGGCGTAA	152
DabiGST7	OP485325	CCAACAACAGATTGACCTTG	GCTCTAATCCAGGCACATT	162
**House keeping gene**				
EF1α1	OP485296	GGTGCGAATACAACAATGG	GTAAGGAAGGTAAGGCTGAA	193
RPS3	MH557092	TGATGAATCTCAGCACACCATAGCA	ACGCCTACACGCTCCGAGAT	256
